# Intumescent Silicate Coatings with the Addition of Alkali-Activated Materials

**DOI:** 10.3390/polym14101937

**Published:** 2022-05-10

**Authors:** Nicoleta Florentina Cirstea, Alina Badanoiu, Aurelian Cristian Boscornea

**Affiliations:** 1 Department of Science and Engineering of Oxide Materials and Nanomaterials, Faculty of Applied Chemistry and Materials Science, University “Politehnica” of Bucharest, Polizu 1–7, 011061 Bucharest, Romania; cirsteanicoleta96@yahoo.com; 2 Department of Bioresources and Polymers Science, Faculty of Applied Chemistry and Materials Science, University “Politehnica” of Bucharest, Polizu 1–7, 011061 Bucharest, Romania; cristian.boscornea@upb.ro

**Keywords:** alkali silicate paint, intumescence, waste glass, slag, alkaline activators, fire behavior

## Abstract

Fireproof inorganic coatings based on sodium silicate solution with intumescent additions were prepared and tested to assess their ability to limit the negative effect of a fire. The intumescent materials were obtained by the alkali activation of waste glass powder (obtained by the grinding of recycled soda-lime culet) and slag (waste resulting from the metallurgical industry). The replacement of talc (used as filler in paint formulation) with the intumescent materials obtained by the alkaline activation of waste glass powder (WGP), determined an increase in the intumescence coefficient (up to 65%) and decreased the activation temperature of this process. To evaluate these coatings’ abilities to prevent or delay the temperature increase in metal structures, the paints were applied on steel plates and tested in direct contact with the flame of a butane burner for 60 min. The coatings prevented the increase in the steel substrate temperature over one considered critical (500°C) for steel mechanical properties; the combination of two coatings, with different intumescence activation temperatures, correlated with the increase in the coating’s thickness, sensibly reduced the rate of temperature increase (up to 75%) in the steel substrate.

## 1. Introduction

Fire is a serious threat to life and property. The release of noxious gases during fires can harm humans and the environment; moreover, for steel structures, the consequence of a fire can be catastrophic, given the fact that steel starts losing its mechanical properties and load-bearing capacity when heated to temperatures above 500–600 °C [1,2,3].

To protect the steel structure and to prolong the fire rating time, intumescent coatings can be used. When the temperature increases, these coatings swell and form a porous layer with low thermal conductivity, which provides a protective barrier and prolongs the time available for the safe evacuation of the building [1,2,4].

Intumescent coatings can be organic (based on polymers) or inorganic (based on alkali silicates) [1,5].

An organic intumescent coating is formulated with a mixture of a binder (epoxy, polyvinyl acetate, polyacrylate, etc.), a carbon donor or a char former (for example, pentaerythritol), an acid donor (ammonium polyphosphate) and a blowing agent [1]; this type of coating can also contain a wide range of flame retardant fillers such as Al(OH)_3_, Mg(OH)_2_, TiO_2_, CaCO_3_, calcium silicates [6], carbon nano tubes [7], graphene [8], modified montmorillonite clay [9] or flame-retardant inorganic fibers (silica, alumina or ceramic fibers) [10], etc. According to Puri and Khanna [1], organic intumescent coatings have numerous advantages such as ease of application, good quality finish and important swelling when exposed to heat. The main disadvantages of these types of intumescent coatings are connected with the release of smoke and toxic fumes (mainly due to the organic components) and, in some cases, the potential displacement of the protective char layer formed during the fire [1,3].

Inorganic intumescent alkali silicate coatings can be applied to a wide range of surfaces such as wood, polymers, metal, concrete, plaster, brick, and glass [1,11,12]. The main advantages of using alkali silicate coatings as passive fire protection are: (i) smaller environmental concerns, i.e., these coatings release water vapor during the intumescence process [1]; (ii) resistance to microorganisms; (iii) good resistance to UV radiation; and (iv) odorless [11]. These paints can be used both outdoors and indoors but, due to their sensitivity to carbon dioxide and moisture, indoor applications are more frequent [1].

Silicate coatings swell when heated due to the release of water vapor and form a porous layer, which can prevent fire propagation. Kazmina et al. [11], developed an environmentally friendly and safe fireproof paint based on a potassium silicate aqueous solution with the addition of a magnesium-containing fire retardant, i.e., brucite—Mg(OH)_2_, magnesite—MgCO_3_, and hydromagnesite—Mg_5_[CO_3_]_4_(OH)_2_.4H_2_O. The best results were obtained for the paint with the hydromagnesite addition, which due to its structural characteristics, exhibits a gradual loss of weight during heating, determined by the gradual water separation and release of carbon dioxide. This contributes to the formation of a foam layer with an uniform and finely porous structure with good fire-retardant characteristics. 

In this study, we assess the possibility of improving the fire behavior of this silicate paint by the addition of intumescent materials obtained by the alkaline activation of waste glass powder [13,14,15,16,17]. The alkaline activation of waste glass powder with a mixture of sodium hydroxide and borax determined the formation of alkali-activated borosilicates inorganic polymers (AABSIPs), which exhibit an important increase in volume (250–430%) when heated at temperatures comprised between 400–600 °C [15,16]. Intumescent alkali-activated materials (AAMs) can be also obtained by the mixing of waste glass powder and slag with NaOH solution; the increase in slag content in the mixture also increases the intumescence temperature, an important volume and porosity increase being assessed for specimens thermally treated at 900 °C [13]. Like hydromagnesite, AABSIPs and AAMs exhibit a continuous weight loss when heated up to 600–900 °C; therefore, in this paper, we present results regarding their influence on the intumescent behavior of an environmentally friendly silicate paint. The coatings with intumescent additions were tested in direct contact with a methane flame or were thermally treated for one hour at different temperatures (600 °C, 700 °C, and 900 °C). The modification of the coating thickness (intumescence factor), as well as the changes in their mineralogical composition after the thermal treatment, were assessed. To evaluate these coatings’ abilities to prevent or delay the temperature increase in metal structures, the paints were applied on steel plates and tested in direct contact with the flame of a butane burner. 

## 2. Materials and Methods

The materials used for the preparation of silicate paints were:

- Sodium silicate solution, from Sigma Aldrich (St. Louis, MO, USA), with a density of 1.39 g/cm^3^ at 20 °C;
- Glycerin p.a., from Comchim SA (Bucharest, Romania), conc. 99.5%;
- Styrene-acrylic dispersion Acronal S562, from BASF (Ludwigshafen, Germany), solid content 49–51%, density 1.04 kg/m^3^, viscosity 800 mPa.s (23 °C, 100 1/s) (DIN EN ISO 3219), particle size range: <0.1–10 µm;
- ZnO p.a. from Merck (Darmstadt, Germany), conc. 99.5%;
- CaCO_3_, OMYACARB 1-VO by OMYA (Oftringen, Switzerland), conc. 98.2%, mean particle size (d50%) 2.2 µm, density 0.4 kg/m^3^;
- Talc (Mg-silicate), Finntalc M15 Mondo Minerals B.V (Amsterdam, The Netherlands), conc. 97%, mean particle size (d50%) 4.5 µm, density 0.3 kg/m^3^;
- Silica fume with particle size of 0.007 microns and surface area of 390 ± 40 m^2^/g, from Sigma Aldrich;
- Graphite powder, ACROS ORGANICS (Geel, Belgium), molecular weight = 12.01 g/mol);
- NaOH p.a. from Merck, conc. > 99%;
- Sodium borate decahydrate (B), p.a. from Sigma Aldrich, conc. > 99.5%;
- Waste glass powder (WGP)—obtained by the grinding of soda-lime glass cullet with a Blaine specific surface of 2820 cm^2^/g;
- Blast furnace slag (S)—waste from metallurgic industry, with a Blaine specific surface area of 3300 cm^2^/g.

The formulation of reference paint was the one proposed by Kazmina et al. [11], with the difference that potassium silicate solution was replaced with sodium silicate solution (Table 1). According to Kazmina et al. [11], talc is used as filler to improve the spreading and coverage and to increase the corrosion resistance of the coating, but it does not participate in the foaming process of silicate paint. Therefore, in the new formulations of paints, talc was substituted with intumescent materials obtained by the alkaline activation of waste glass powder.

The intumescent material GZ was obtained by the mixing of waste glass powder with NaOH solution (WGP:S:NaOH = 1:0.3:0.1 wt.; water to solid ratio 0.44) and GB by the mixing of waste glass powder with borax and NaOH solution (WGP:B:NaOH = 1:0.9:0.24 wt.; water to solid ratio 0.34). GZ paste was cured for 4 h in the oven at 60 °C and then in air at 20 ± 2 °C and GB paste was cured at 20 ± 2 °C; after 14 days the pastes were ground in a ball mill and the resulting powders were analyzed by laser granulometry; GZ powder has median volume diameter d(0.5) = 14.15 microns and GB powder has median volume diameter d(0.5) = 116.37 microns. 

Table 1 presents the compositions of the studied paints; as can be seen, talc was replaced with GZ, GB, and a mixture of GB and graphite (GBC). The use of graphite as admixture was selected based on the literature data [18,19].

The components were mixed using a laboratory high-speed Stirrer 492-I by Ericksen GmbH & Co. (Hemer, Germany) at 3000 rpm for 60 min and the obtained paints were stored in tight closed containers.

**Figure 1:**
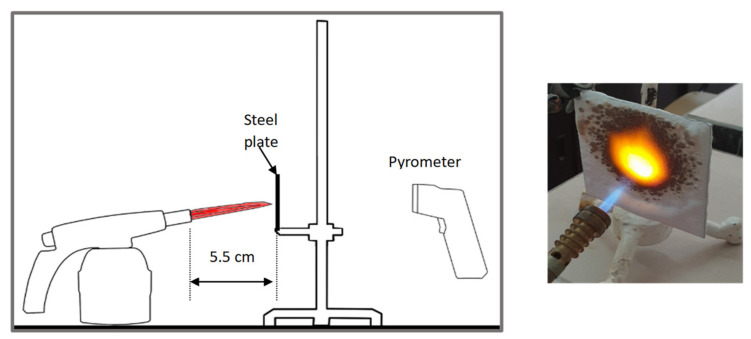
Fire test set up.

The paints were applied in three consecutive layers on steel plates (100 × 100 × 3 mm —length × width × height). The average thickness of the coatings, after drying, was 0.82 mm for S_GZ, 0.89 mm for S_GB and 0.52 mm for S_GBC. Additionally, consecutive layers of S_GZ and S_GB paints were applied on a steel plate; the thickness of this coating was 1.04 mm. The coated steel plates were put in direct contact with a butane flame (using a torch burner). The test setup is presented in Figure 1. On the opposite side of the coated one (denominated as “cold” face), the temperature was measured every 60 s using a pyrometer. The accuracy of pyrometer was ±1% from the recoded value + 1 °C [14].

In order to assess their fire behavior, the paints were dried in air until a rigid material was obtained; this material was cut into square plates (20 mm × 20 mm × 3 mm—length × width × height) and treated in an electric oven at 600 °C, 700 °C and 900 °C, for 60 min (heating rate = 10 °C/min). 

The intumescence coefficient was calculated with the formula [11]:

K = [(h_1_ − h_0_)/h_0_] × 100 (%) (1)

where h_0_ = initial height (thickness) of the specimen; h_1_ = specimen height (thickness) after thermal treatment. 

Additionally, specimens with 20 mm × 10 mm × 3 mm (length × width × height) were kept in direct contact with a methane flame (Bunsen burner) for 2 min, and the changes visually assessed were recorded. 

The X-ray diffraction (XRD) analyses were performed with an XRD 6000 Shimadzu diffractometer (Shimadzu, Kyoto, Japan), CuKα (λ = 1.5406 Ȧ) radiation with a scanning speed of 2°/min.

## 3. Results and Discussions

**Figure 2:**
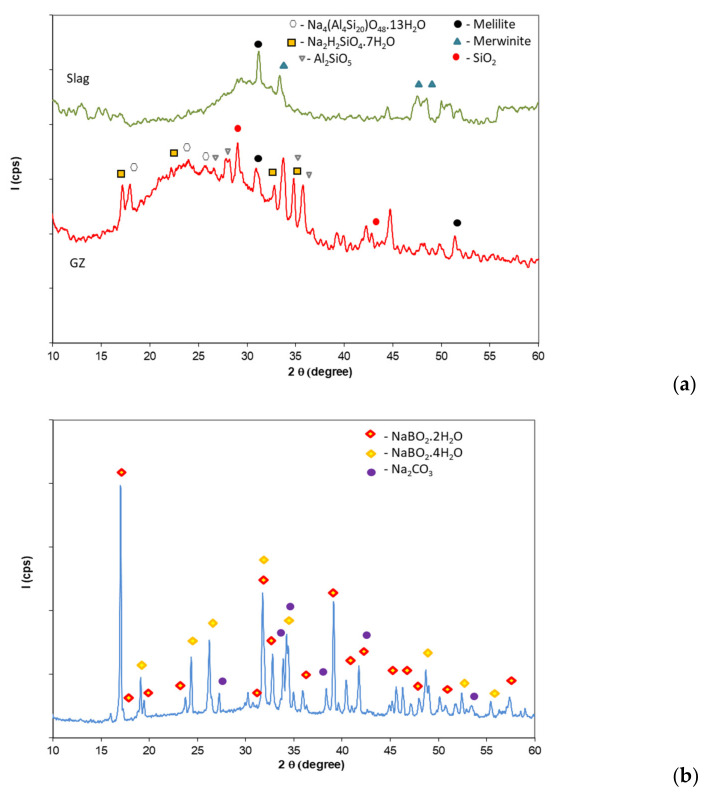
XRD patterns of: (**a**) slag and alkali-activated mixture of waste glass and slag (GZ); (**b**) waste glass activated with a mixture of NaOH and borax (GB).

The mineralogical compositions of the intumescent powders used as fillers for the manufacture of intumescent silicate paints are presented in Figure 2.

The slag used in this research contains over a 90% vitreous phase and a low amount of merwinite and mellite minerals. The XRD patterns of the alkali-activated waste glass and slag mixture (GZ—Figure 2a) also show the presence of a vitreous phase (wide halo at 2θ = 15–35 degrees) along with small amounts of sodium silicate and sodium silicate aluminate hydrates. The alkaline activation of waste glass powder with a mixture of NaOH and borax (GB—Figure 2b) determined the formation of sodium metaborate hydrates [15,16]; the presence of a small amount of Na_2_CO_3_ is most probably due to the partial carbonation of unreacted NaOH. 

The behavior of the silicate coatings, when put in direct contact with the methane flame (Bunsen burner), is presented in Table 2. 

As can be seen from Table 2, the reference silicate coating (Ref) starts to emit a white gas after 30 s from the moment when it is put in direct contact with the flame; the material turns gray, and its volume and porosity increase. The paints modified with the intumescent materials GZ and GB also exhibit an increase in volume and porosity, but gas emissions are not visually detected as in the Ref case. 

The paint with GB and graphite additions (GBC) starts to change its color from black to white from the first moment when put in contact with the flame; this process is very fast (20 s) and causes a certain increase in volume; this white layer continues to expand, and the color gradually turns gray. 

**Figure 3:**
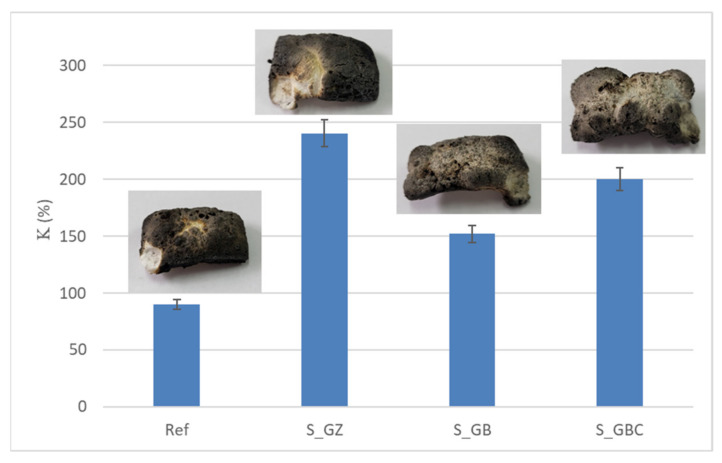
Visual aspect and intumescence coefficient (K) after 2 min of direct contact with the flame.

The replacement of talc (used as filler in paint formulation) with the intumescent materials obtained by the alkaline activation of waste glass powder (WGP) determined an increase in the intumescence coefficient, the highest values being recorded for S_GZ and S_GBC coatings (Figure 3).

**Figure 4:**
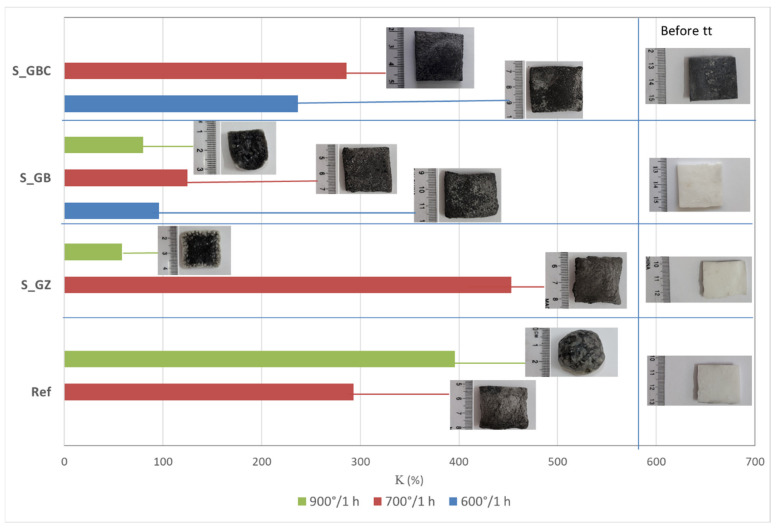
Visual aspect and intumescence coefficient (K) after 60 min of thermal treatment (tt) at different temperatures.

The intumescence coefficients of the studied coatings after thermal treatment for one hour at different temperatures are presented in Figure 4. As can be seen, the thermal treatment of the reference (Ref) at 700 °C and 900 °C determines an important increase in the volume and porosity. The replacement of talc with the GZ, GB, and GB + graphite, in the composition of the silicate paints, decreases the temperature for which the highest intumescence coefficient is recorded; for the S_GZ coating, the highest expansion is recorded at 700 °C and the further increase in the temperature to 900 °C determines the partial melting and deformation of the specimen. For the S_GB paint, the highest value of intumescence coefficient is recorded at 700 °C and is much smaller compared with the one recorded for S_GZ. The addition of graphite in S_GBC increases the intumescence coefficient, as compared with S_GB for both thermal treatment temperatures—600 °C and 700°C. For all studied coatings, the thermal treatment at 900 °C determined the partial melting of the materials and specimen deformation, more important for those with intumescent additions. 

**Figure 5:**
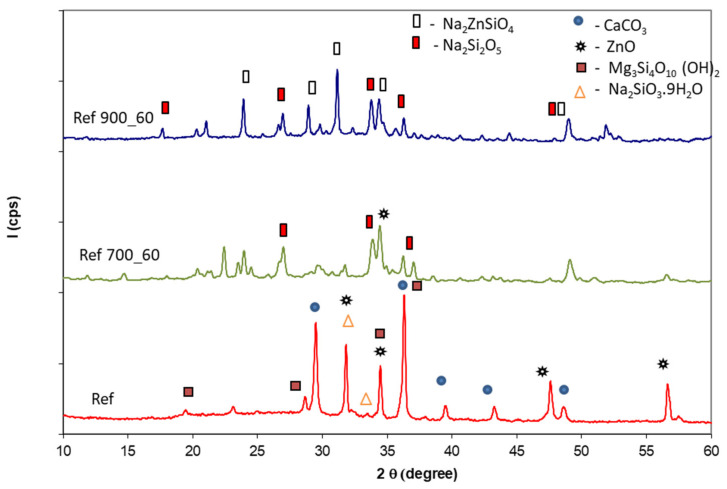
XRD patterns of reference coating before and after thermal treatment at 700 °C and 900 °C for 60 min.

**Figure 6:**
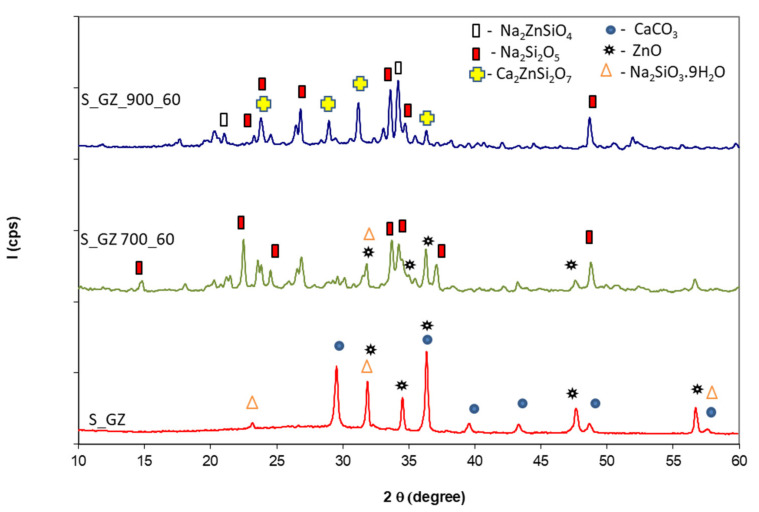
XRD patterns of the coating with GZ addition before and after thermal treatment at 700 °C and 900 °C for 60 min.

**Figure 7:**
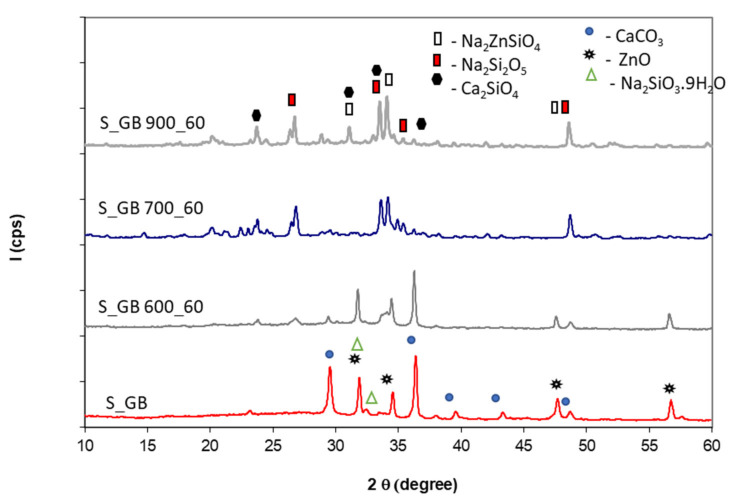
XRD patterns of the coating with GB addition before and after thermal treatment at 600 °C, 700 °C and 900 °C for 60 min.

**Figure 8:**
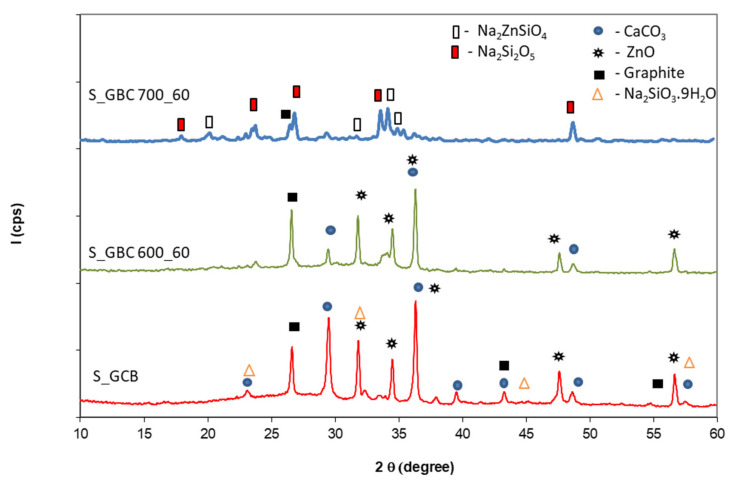
XRD patterns of the coating with GB and graphite addition before and after thermal treatment at 600 °C and 700 °C for 60 min.

Figure 5, Figure 6, Figure 7 and Figure 8 show XRD patterns of the Ref, S_GZ, S_GB, and S_GBC coatings before and after thermal treatment at different temperatures (600 °C, 700 °C, and 900 °C). 

As can be seen, the XRD peaks specific to the sodium silicate hydrate (Na_2_SiO_3_∙9H_2_O), CaCO_3_, ZnO, and talc, are present on the XRD patterns of the reference coating, before thermal treatment (Figure 5). The thermal treatment at 700 °C for 60 min determines the formation of Na_2_SiO_5_ [20], and a further increase in the thermal treatment temperature at 900 °C determines the formation of Na_2_ZnSiO_4_ [21].

On the XRD patterns of the S_GZ coating, one can also assess the presence of sodium silicate hydrate, CaCO_3_ and ZnO (Figure 6). Due to the low degree of crystallinity of GZ and its small amount, this component cannot be detected on XRD patterns of the S_GZ coating. The thermal treatment of this coating also leads to the crystallization of sodium silicate (Na_2_Si_2_O_5_) and the formation of sodium zinc silicate. In this system is also possible the formation of hardystonite (Ca_2_ZnSi_2_O_7_) [22]. 

For the composition with GB (i.e., with boron content)—Figure 7, the thermal treatment also determines the crystallization of Na_2_SiO_5_, the formation of sodium zinc silicate and at higher temperature (900 °C) due to the fluxing effect of boron [23] the formation of Ca_2_SiO_4_ is also possible. 

The presence of graphite in the GBC coating does not modify the nature of the phases detected by XRD (Figure 8); the main compounds assessed in the sample thermally treated at 700 °C are Na_2_Si_2_O_5_ and Na_2_ZnSiO_4_. The decrease in the graphite content for the specimen treated at 700 °C for 60 min as compared with reference and the sample treated at 600 °C can be observed. This can be explained by the graphite oxidation with CO_2_/CO generation, a process that occurs between 600–800 °C for graphite powder with particle sizes smaller than 20 microns [24].

In order to assess the efficiency of this type of coating to prevent the increase in the temperature of a metallic structure, the steel plates with intumescent coatings were put in direct contact with a butane flame. The visual aspect of the steel plates before, during and after the test are presented in Table 3. 

As can be observed, a higher expansion of the coating was achieved for the reference and S_GZ paints. For all coatings, in the area where the flame was in direct contact with the material for one hour, cracking, partial melting, and delamination of this layer occurred; this process is more important for the coatings with GB and GBC additions, which, according to the previously presented data, tends to melt at lower temperatures as compared with the GZ and reference coating. 

Interestingly to note, for the steel plate covered with two different coatings (first layer of S_GZ and the second of S_GB), when put in contact with the flame, the superior coating first exhibits intumescent behavior (swelling) and then melts, but the inner layers remain well adhered to the surface of the steel plate, thus preventing the temperature increase in the substrate in the future.

**Figure 9:**
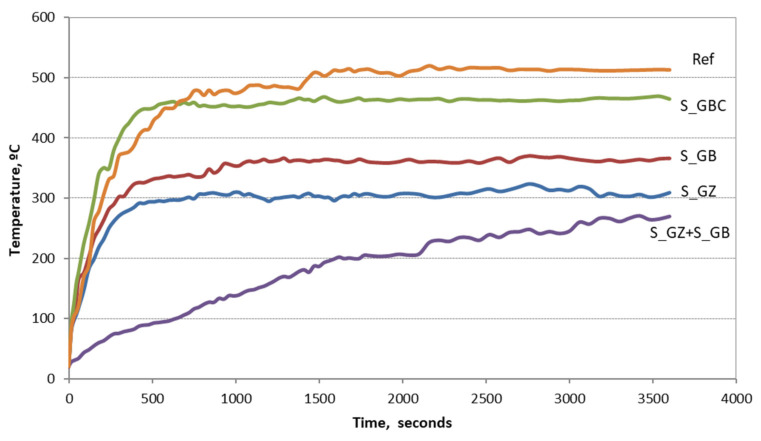
The evolution of the temperature of “cold” face vs. time for the plates covered with studied silicate coatings.

Figure 9 shows the evolution vs. time of the temperature of the support, measured on the opposite side of the metal plates coated with the studied paints.

A fast increase in the “cold” face temperature can be observed when the plates covered with reference coating (Ref) are put in direct contact with the butane flame. This could be due to the rapid increase in coating thickness, quickly followed by the melting and cracking, therefore the flame can easily penetrate the unprotected surface of the steel plate. The higher rate of temperature increases for the steel plate covered with the S_GBC coating, correlated with the initial whitening of the coating, is most probably due to the rapid graphite oxidation, which is an exothermic effect [25]. The addition of GB and GZ slightly decreased the rate of temperature increase and the plateau temperature achieved for these specimens is much smaller as compared with those achieved for the reference and S_GBC. The best behavior was achieved for the steel plate covered with two different coatings (S_GZ and S_GB), i.e., the lowest rate for the increase in “cold” face temperature and the lowest temperature of the support after one hour of contact with the flame. This behavior is explained both by the increase in the coating’s thickness, as well as by the combination of the S_GB paint, which has a lower activation temperature of the intumescent process, and the S_GZ paint, with a higher activation temperature. 

For all studied paints, the adhesion to the steel substrate was very good, and the coatings remained attached to the steel plate in the adjacent zones where the coating was in direct contact with the flame. 

## 4. Conclusions

Fireproof inorganic paints based on sodium silicate solution with intumescent additions were obtained and tested in this research. The intumescent materials were obtained by the alkali activation of waste glass powder (obtained by the grinding of recycled soda-lime culet) and slag (waste resulting from the metallurgical industry). 

The replacement of talc (used as filler in paint formulation) with the intumescent materials obtained by the alkaline activation of waste glass powder (WGP) determined an increase in the intumescence coefficient (up to 65%) and decreased the activation temperature of this process.

To assess the efficiency of these types of coatings to prevent the increase in the temperature of a metallic structure, steel plates were coated with the studied paints and put in direct contact with a butane flame for 60 min. The coatings prevented the increase in the steel substrate temperature over one considered critical (500 °C) for the steel’s mechanical properties. The rate of the temperature increase was smaller for the coatings with intumescent fillers as compared with the reference; moreover, the successive application of two types of coatings (containing the two studied intumescent additions) and the increase in the coating thickness efficiently limited the increase in the steel substrate temperature. 

These results confirm the possibility of obtaining environmentally friendly intumescent silicate paints by the valorization of two types of wastes (waste glass powder and slag).

## Figures and Tables

**Table 1:** Compositions of silicate paints with/without intumescent material.

Paint	Sodium Silicate %	Glycerin %	Styrene-acrylic Dispersion %	ZnO %	CaCO_3_ %	SiO_2_ %	Talc %	GZ %	GB %	Graphite %
Ref	69.7	5.0	5.0	6.1	8.6	0.1	5.5	-	-	-
S_GZ	69.7	5.0	5.0	6.1	8.6	0.1	-	5.5	-	-
S_GB	69.7	5.0	5.0	6.1	8.6	0.1	-	-	5.5	-
S_GBC	69.7	5.0	5.0	6.1	8.6	0.1	-	-	3.0	2.5

**Table 2:** Visual aspect of samples during direct contact with the flame.

	33 s	50 s	1 min 42 s	2 min
Ref	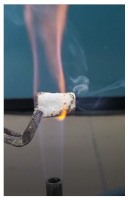	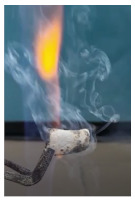	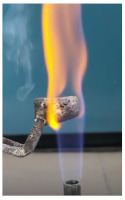	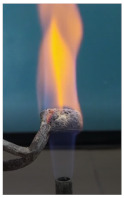
S_GZ	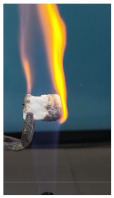	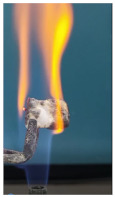	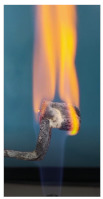	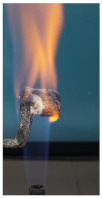
S_GB	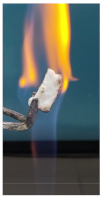	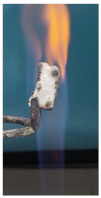	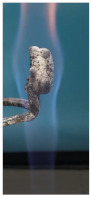	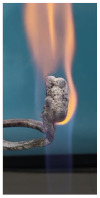
S_GBC	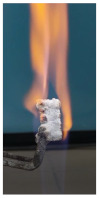	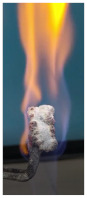	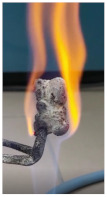	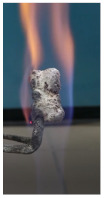

**Table 3:** Visual aspect of metal plates coated with intumescent silicate paints, before, during and after direct contact with the flame.

	Ref	S_GZ	S_GB	S_GBC	S_GZ + S_GB
Before the test	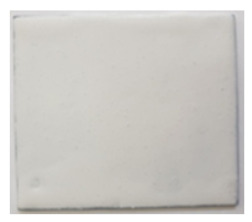	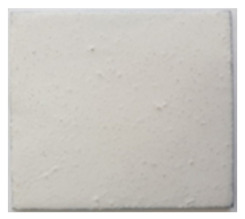	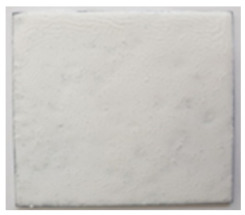	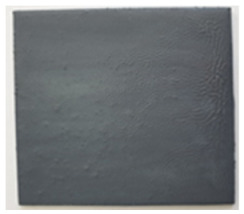	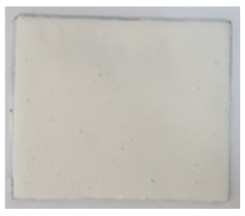
During the test	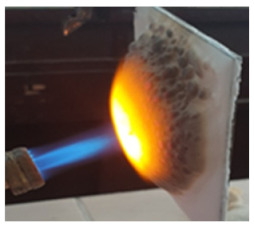	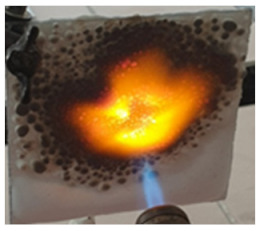	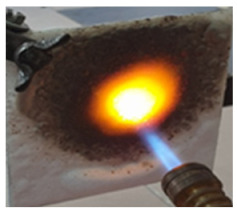	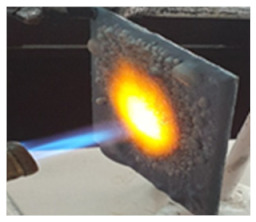	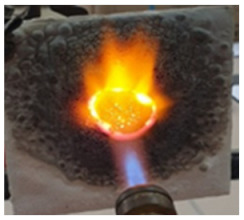
After the test	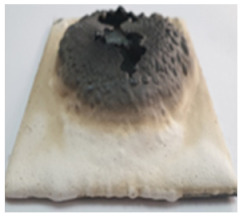	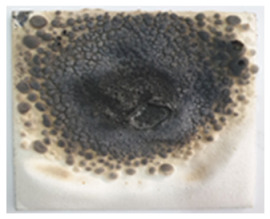	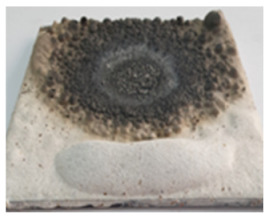	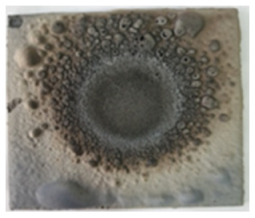	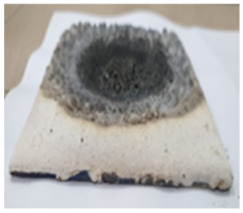

## Data Availability

Not applicable.
